# Beta-Catenin Signaling Plays a Disparate Role in Different Phases of Fracture Repair: Implications for Therapy to Improve Bone Healing

**DOI:** 10.1371/journal.pmed.0040249

**Published:** 2007-07-31

**Authors:** Yan Chen, Heather C Whetstone, Alvin C Lin, Puviindran Nadesan, Qingxia Wei, Raymond Poon, Benjamin A Alman

**Affiliations:** 1 Program in Developmental and Stem Cell Biology, the Hospital for Sick Children, University of Toronto, Toronto, Canada; 2 Division of Orthopaedic Surgery, Department of Surgery, University of Toronto, Toronto, Canada; Shriner's Hospital Portland, United States of America

## Abstract

**Background:**

Delayed fracture healing causes substantial disability and usually requires additional surgical treatments. Pharmacologic management to improve fracture repair would substantially improve patient outcome. The signaling pathways regulating bone healing are beginning to be unraveled, and they provide clues into pharmacologic management. The β-catenin signaling pathway, which activates T cell factor (TCF)-dependent transcription, has emerged as a key regulator in embryonic skeletogenesis, positively regulating osteoblasts. However, its role in bone repair is unknown. The goal of this study was to explore the role of β-catenin signaling in bone repair.

**Methods and Findings:**

Western blot analysis showed significant up-regulation of β-catenin during the bone healing process. Using a β-Gal activity assay to observe activation during healing of tibia fractures in a transgenic mouse model expressing a TCF reporter, we found that β-catenin-mediated, TCF-dependent transcription was activated in both bone and cartilage formation during fracture repair. Using reverse transcription-PCR, we observed that several WNT ligands were expressed during fracture repair. Treatment with DKK1 (an antagonist of WNT/β-catenin pathway) inhibited β-catenin signaling and the healing process, suggesting that WNT ligands regulate β-catenin. Healing was significantly repressed in mice conditionally expressing either null or stabilized β-catenin alleles induced by an adenovirus expressing Cre recombinase. Fracture repair was also inhibited in mice expressing osteoblast-specific β-catenin null alleles. In stark contrast, there was dramatically enhanced bone healing in mice expressing an activated form of β-catenin, whose expression was restricted to osteoblasts. Treating mice with lithium activated β-catenin in the healing fracture, but healing was enhanced only when treatment was started subsequent to the fracture.

**Conclusions:**

These results demonstrate that β-catenin functions differently at different stages of fracture repair. In early stages, precise regulation of β-catenin is required for pluripotent mesenchymal cells to differentiate to either osteoblasts or chondrocytes. Once these undifferentiated cells have become committed to the osteoblast lineage, β-catenin positively regulates osteoblasts. This is a different function for β-catenin than has previously been reported during development. Activation of β-catenin by lithium treatment has potential to improve fracture healing, but only when utilized in later phases of repair, after mesenchymal cells have become committed to the osteoblast lineage.

## Abstract 2

In a study in mice Benjamin Alman and colleagues show that β-catenin functions differently in different stages of fracture repair; moreover, activation of β-catenin by lithium improves fracture healing when used in later phases of repair.

## Editors' Summary

**Background.:**

Most people break at least one bone during their life. If the damaged bone is immobilized with a plaster cast or with metal plates and pins, most fractures heal naturally and quickly. Soon after a bone is damaged, cells called pluripotent mesenchymal cells collect at the injury site. Here, they multiply and change (differentiate) into osteoblasts (cells that make bone) and chondrocytes (cells that make cartilage, the dense connective tissue that covers joints). Osteoblasts and chondrocytes mend the fracture by making new bone, a process called ossification. Bone healing involves two types of ossification. In intramembranous ossification, mesenchymal cells and osteoblast progenitor cells make bone directly, forming a hard “callus” within the fracture. In endochondral ossification, mesenchymal cells differentiate into chondrocytes and make cartilage at the fracture site, which osteoblasts turn into bone. Finally, the bone made by both types of ossification is remodeled so that it closely resembles the damaged bone's original shape and strength.

**Why Was This Study Done?:**

Unfortunately, fractures do not always heal efficiently. If healing is delayed, additional surgery may be needed to repair the break. But surgery can be risky, so drug-based ways of encouraging bone repair would be very useful. To develop such treatments, researchers need to understand what controls the differentiation and activity of osteoblasts and chondrocytes during normal healing. In this study, the researchers have investigated the role of the β-catenin signaling pathway in bone repair. This pathway regulates bone formation during embryonic development, a process that closely resembles bone healing. β-catenin is usually degraded rapidly in cells. However, if a member of a particular family of proteins known as the WNT family binds to a WNT receptor on the surface of a cell, β-catenin moves into the cell's nucleus where it interacts with a protein called T cell factor (TCF). This interaction activates the transcription (the copying of DNA into messenger RNA, which is used to make proteins) of numerous genes and alters the behavior of the cell.

**What Did the Researchers Do and Find?:**

The researchers first measured β-catenin levels in mouse and human bones. In both species, much more β-catenin was made in bones undergoing repair than in intact bones. Then they studied TCF reporter mice—animals in which TCF controls the expression of a marker gene. β-catenin-mediated TCF-dependent transcription, they report, was activated during both bone and cartilage formation after a fracture in these mice. Next, the researchers made mice that could be induced to express an inactive form of β-catenin or a stabilized (permanently active) form of β-catenin in all the cells in a bone fracture. Expression of inactive β-catenin slowed the rate of healing but, unexpectedly, so did expression of stabilized β-catenin. Osteoblast-specific expression of inactive β-catenin also delayed bone healing, whereas osteoblast-specific expression of stabilized β-catenin enhanced the process. Finally, treatment of wild-type mice with lithium (which prevents the degradation of β-catenin) enhanced bone healing if given after a fracture, but interfered with it if given before.

**What Do These Findings Mean?:**

These findings indicate that β-catenin signaling (which, the researchers show, is mainly activated by WNT signaling) has different effects at different stages of bone repair. Early in the process, it controls the ratio of osteoblasts and chondrocytes made from the pluripotent mesenchymal cells. Consequently, too much or too little β-catenin interferes with bone healing at this stage. Later on, β-catenin promotes the differentiation of osteoblasts and enhances their ability to make bone, and so too little β-catenin at this stage prevents healing, whereas increased β-catenin levels stimulate healing. These findings need to be confirmed in people before testing agents that affect β-catenin signaling for their effects on human bone healing. Nevertheless, the researchers' final discovery that lithium improves bone healing if given at the right time is particularly encouraging; lithium is widely used to treat one form of depression so could be readily tested in clinical trials.

**Additional Information.:**

Please access these Web sites via the online version of this summary at http://dx.doi.org/10.1371/journal.pmed.0040249.

- MedlinePlus encyclopedia contains pages on broken bones and on bone fracture repair (in English and Spanish)
- Wikipedia has pages on bone fracture and on bone healing (note: Wikipedia is a free online encyclopedia that anyone can edit; available in several languages)
- The UK National Health Service Direct encyclopedia provides pages on broken bones
- Animations of intramembranous and endochondral ossification are available from the Ministry of Advanced Education, Training and Technology, Province of British Columbia, Canada
- The American Academy of Orthopedic Surgeons has an informative discussion of fractures
- The Hospital for Sick Children in Toronto (where the authors of this study are affiliated) has a Web site called SickKids, which contains a page on child physiology, including diagrams of bone development

## Introduction

Fracture healing is a complex regenerative process initiated in response to injury, which in the optimal case results in restoration of skeletal function. In the initial phase of fracture repair, undifferentiated mesenchymal cells aggregate at the site of injury, proliferate, and differentiate, presumably in response to growth factors produced by the injured tissues [1]. This process involves both intramembranous and endochondral ossification. Intramembranous ossification involves the formation of bone directly from committed osteoprogenitor cells and undifferentiated mesenchymal cells that reside in the periosteum, resulting in hard callus formation [2,3]. During endochondral ossification, mesenchymal cells differentiate into chondrocytes, producing cartilaginous matrix, which then undergoes calcification and eventually is replaced by bone. The formation of primary bone is followed by extensive remodeling until the damaged skeletal element regains original shape and size. These processes are reminiscent of embryonic bone development, suggesting that fracture repair recapitulates normal bone development [4–6]. When fracture healing is impaired, osteoblastic differentiation is inhibited, and undifferentiated mesenchymal tissue remains at the fracture site. In patients, this outcome results in delayed union, or nonunion, usually requiring additional surgery for successful fracture healing. In certain surgeries, such as spinal fusion surgery or total joint replacement, a lack of bone ingrowths results in a failed outcome and the need for additional surgery. A pharmacologic adjunct to improve bone healing has the potential to avoid the need for additional surgery in these cases, improving patient outcomes.

β-catenin signaling has emerged as a key regulator of embryonic bone development [7,8]. Secreted WNT ligands and antagonists of WNT signaling bind to the transmembrane receptors including low-density lipoprotein jureceptor-related protein 5/6 (LRP5/6) and frizzled (FZ) to regulate the WNT pathway. WNT signaling activation produces a cytoplasmic signaling cascade leading to the transcriptional regulation of gene expression, cytoskeletal reorganization, and calcium flux [9]. Signaling through the canonical WNT pathway is initiated by the binding of appropriate WNT ligands to the FZs and LRP5/6 co-receptor. In the absence of appropriate WNT ligands, β-catenin is targeted for phosphorylation, ubiquitination and proteosomal degradation by a multi-protein complex comprising glycogen synthase kinase 3 beta (GSK3β), adenomatous polyposis coli, and axin [10–12]. In the presence of an appropriate WNT ligand, this multi-protein complex is dissociated by dishevelled (DVL), an intracellular mediator that plays a central role in transducing the signal from the receptor complex, leading to the activation of β-catenin [13]. Hence, β-catenin cannot be targeted for degradation and translocates to the nucleus, where in concert with members of the T cell factor/lymphoid enhancer factor (TCF/LEF) family, activates the transcription of a wide range of genes. During embryonic skeletogenesis, inhibition of β-catenin signaling can block osteoblast differentiation and shift pluripotent mesenchymal cells to develop a chondroblastic phenotype [14,15]. β-catenin signaling also can regulate bone mass, and its activation results in increased bone density [16–18].

Although the histology and general mechanism of fracture repair are clear and well established, much remains to be understood about the process of bone healing, particularly at the molecular signaling level. Based on a microarray analysis, it was found that WNT pathway members are expressed during fracture repair [19]. DVL is expressed during fracture repair and when blocked using small interfering RNAs (siRNAs), inhibits both proliferation and differentiation of chondrocytes [20]. *WNT1-induced secreted protein 1 (WISP1),* a WNT target gene, is also expressed during fracture healing, and overexpression of this gene increases proliferation but decreases the differentiation in a chondrocytic cell line [21]. We previously reported that β-catenin regulates wound size during dermal repair [22], and that oral lithium treatment could be used to activate β-catenin signaling and to increase the size of healing cutaneous wounds [23]. Based on these findings, it is likely that β-catenin plays a role in fracture repair. In this study, we aimed at investigating how β-catenin signaling functions during fracture healing, and to test for the effect of lithium treatment. Since lithium is a pharmacologic agent already approved for use in patients, this approach could be developed into a novel therapy to improve bone healing.

## Methods

### Ad-DKK1 and Ad-Cre

Dickkopf 1 (DKK1) is a secreted protein that binds to LRP5/6 and Kremen proteins, blocking canonical WNT signaling. An adenovirus harboring murine *DKK1* cDNA with a C-terminal His6 and Flag epitope tags (Ad-DKK1) was generated and utilized as previously described [24]. Cre recombinase expression was driven by a CMV promoter in an adenovirus construct (Ad-Cre) generated and utilized as previously described [22]. The same adenovirus expressing GFP was utilized as a control. Ad-Cre's ability to cause recombination was assessed using reporter mice as we previously described [22]; it resulted in recombination in roughly 75% of cells.

### Transgenic Mice

*Catnb^tm2Kem^* mice [25] possess loxP sites located in introns 1 and 6 of the gene encoding β-catenin, resulting in a null allele when treated with a Cre recombinase. *Catnb^lox(ex3)^* mice [26] contain loxP sequences flanking exon 3, and when subjected to Cre recombinase, results in the expression of a fully functional but stabilized β-catenin protein. TCF reporter mice [22] contain a *LacZ* gene downstream of a *c-fos* minimal promoter and three consensus TCF-binding motifs. Mice expressing osteoblast-specific β-catenin null alleles, *α1(I)-Catnb^null^,* were generated by mating *α1(I)-Cre* mice [27] with *Catnb^tm2Kem^* animals, and mice expressing osteoblast-specific β-catenin stabilized alleles, *α1(I)-Catnb^stab^,* were generated by mating *α1(I)-Cre* mice with *Catnb^lox(ex3)^* mice. *α1(I)-Cre* mice express the Cre recombinase at high levels in cells committed to the osteoblast lineage. Activation of the conditional alleles was confirmed by Western blot analysis and by using a Cre-reporter mouse as previously reported [25–27].

### Generation of Fractures

All animal procedures were reviewed and approved by the animal care committee of the Hospital for Sick Children in Toronto. Male mice aged 12 wk were utilized for this study. A stabilized fracture was generated as previously reported with minor modifications [28]. Briefly, a small incision was made on the dorsolateral side of the thigh and was extended over the knee region. A longitudinal incision was made in the patellar tendon, and a 0.5 mm hole was drilled above the tibia tuberosity. Intramedullary fixation was made by placing an Anticorro insect pin (Fine Science Tools, http://www.finescience.com/) in the marrow space. A fracture was then made by cutting the shaft of tibia. Previous data show that a fracture generated in this manner heals through both endochondral and intramembranous ossification [28,29]. For virus treatment, Ad-DKK1, Ad-Cre, or Ad-GFP at a dosage of 10^9^ pfu per mouse was mixed with Matrigel (BD Biosciences, http://www.bdbiosciences.com/) and injected into the fracture site using a microsyringe. For mice treated with lithium, sterile 0.6 M LiCl (or 0.6 M NaCl as a control) was added to the drinking water leading to a final concentration of 0.02 M. This oral dosage (∼200 mg/kg per day, a dose giving plasma levels comparable with level used to treat humans with bipolar illness), previously reported to be effective in mice [30,31], was administered either 2 wk prior to, or 4 d after fracture. The animals were allowed free, unrestricted weight bearing in cages after recovery from anesthesia. At different time points (3, 4, 7, 14, and 21 d) after the fracture, mice were sacrificed and fracture callus was harvested for further analysis. At least five animals at each time point for each experimental condition were studied.

### Evaluation of Fracture Healing

Animals were examined using Faxitron MX20 X-ray system (Faxitron X-ray Corporation, http://www.faxitron.com/) for radiographic appearance, and for quantitative analysis of the bone mineral density at the fracture site using the Lunar PIXI Small Animal Bone Densitometer (http://www.gehealthcare.com/). For histological analysis, the tissue at the fracture site was harvested, fixed in 4% paraformaldehyde, decalcified in 20% EDTA (pH 7.4), and embedded in paraffin. Sections 10 μm thick were prepared and stained with hematoxylin–eosin (HE) and safranin O (SO). For histomorphometric measurements, an average of ten tissue sections was used to determine callus parameters. An analysis image window, measuring 1.6 mm^2^, was established for evaluation of cartilage or bone volume as a percentage of total callus tissue volume, trabecular thickness (μm), trabecular number (per mm), and trabecular separation (μm). The radiographs, bone mineral density, and histological parameters of the cartilage and bone were evaluated in a blinded manner by an independent investigator. At least four animals were analyzed for each group for histological and radiographic parameters. For *LacZ* staining, samples were fixed in 4% paraformaldehyde and stained as previously described [32]. Samples were then decalcified in 20% EDTA, and embedded in paraffin. Sections were counterstained with Neutral Red.

### Reverse Transcription PCR and Western Blot

Total RNA and protein were isolated from the fracture site, which encompassed the entire callus and less than 2 mm of adjacent bone. This area corresponds to approximately those bounded by the lines indicating the proximal and distal aspect of the fracture callous in the low-magnification histological images of the fractures. Quantitative reverse transcription PCT (RT-PCR) was performed as previously described [32], with primers listed in Table S1. Expression was compared to β-2 macroglobin as a “housekeeping” control. All experiments were performed in triplicate. Western blot analysis was performed using select antibodies detected using horseradish peroxidase-conjugated secondary antibody and the ECL chemiluminescence detection system (Amersham, http://www.gelifesciences.com/). Relative expression levels were quantified by densitometry using AlphaEaseFC software (Alpha Innotech, http://www.alphainnotech.com/). Specific antibodies utilized in this work are listed in the Table S2.

### Human Fracture Sample

Samples from healing fractures from three patients undergoing corrective surgery for a fracture malunion, two performed 3 wk following the initial fracture and one performed 3 mo following the initial fracture, were cryopreserved as soon as possible after surgery. The patients gave written informed consent to remove fracture samples. These samples were analyzed by Western blot in an identical manner to the mouse studies.

### Statistical Analyses

Data were expressed as mean ± standard deviation. Statistical differences were calculated using Student *t* test. A minimum of four animals were analyzed for each group for histological and radiographic parameters. A *p*-value below 0.05 was considered statistically significant.

## Results

### β-Catenin Signaling Is Activated during Fracture Healing

To determine the protein level of β-catenin during fracture healing, we performed Western blot analysis on protein from the healing callus at different time periods in TCF reporter mice with stabilized tibia fractures. Previous studies show that these murine fractures heal through intramembranous and endochondral ossification by a mechanism similar to that in normal human fracture repair [28,29].

**Figure 1:**
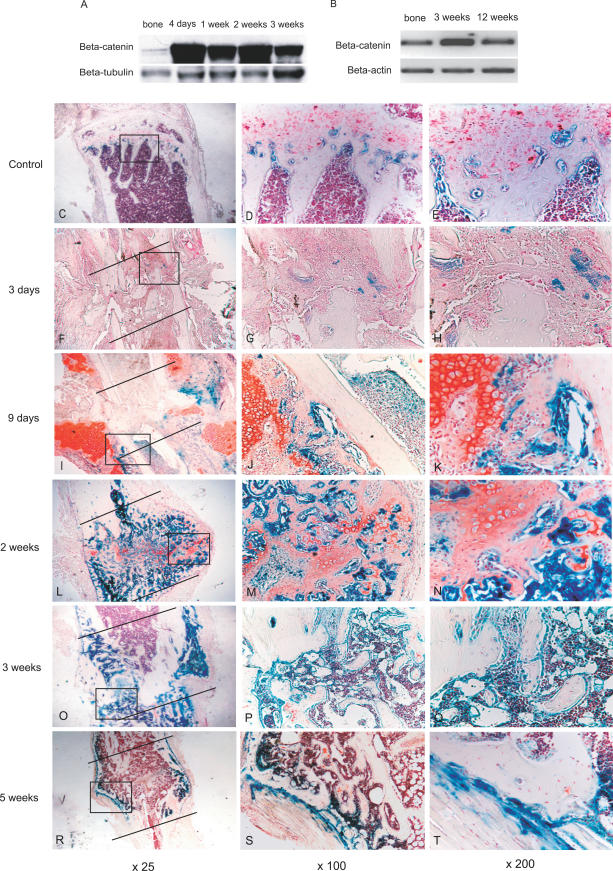
β-Catenin-Mediated TCF-Dependent Transcription Is Activated during Fracture Healing (A) Western blot analysis at different time points shows that β-catenin is elevated throughout healing in mice. (B) β-catenin is also increased during human fracture healing. (C–T) *LacZ* staining for TCF-dependent transcriptional activity in intact tibia (C–E); at 3 d following the tibia fracture (F–H); at 9 d following the fracture (I–K); at 2 wk following fracture (L–N); at 3 wk following fracture (O–Q); and at 5 wk following fracture (R–T). Images on the left (C, F, I, L, O, and R), at 25×, show the entire fracture callus (lines show the proximal and distal aspect of the fracture callus); in the center (D, G, J, M, P, and S) are 100× magnifications of areas shown in the boxes in the lower-magnification images; and on the right (E, H, K, N, Q, and T), the same images are magnified to 200×. TCF-dependent transcriptional activity was maintained at a very low level in osteoblasts near the growth plate in normal intact tibia, and there was no positive staining in cells surrounding the mature bone tissue. Three days following fracture, very weak *LacZ* (barely detectable) staining was detected in mesenchymal tissues at the fracture site. Nine days following fracture, positive staining was evident mainly in cells surrounding cartilage matrix and osteoblasts along the trabeculae and periosteum. Chondrocytes and prehypertrophic chondrocytes also stained at 2 wk after the fracture. Osteoblasts consistently displayed strong staining signals at 2 and 3 wk following fracture. At the 5 wk time point, *LacZ* staining occurred mainly in osteoblasts in the periosteum either next to, or farther away from, the fracture site.

As shown in Figure 1A, β-catenin protein level is low in intact bone tissue, but is highly expressed during the entire period of fracture repair. Data from the human fracture samples showed a similar pattern (Figure 1B), with high levels of β-catenin, more than twice that of intact bone, in the fracture callus 3 wk after the initial fracture, but low levels 3 mo following the initial injury.

Using TCF reporter mice, we observed in which cells β-catenin mediated TCF-dependent transcription was activated during the fracture process. In the intact tibia, there was only weak *LacZ* staining signal in osteoblasts near the growth plate, and there was no positive staining in cells surrounding the mature bone (Figure 1C–1E). At 3 d after the fracture, undifferentiated fibroblast-like mesenchymal cells filled the fracture gap, and there was only small amount of *LacZ* staining (Figure 1F–1H).

By 9 d following the fracture, most of the callus was composed of cartilage. Some cartilage cells exhibited a hypertrophic phenotype and woven bone was also present. Cells surrounding the cartilaginous matrix showed strong *LacZ* staining. Chondrocytes also displayed staining signal, and the staining intensity was greatly decreased in prehypertrophic chondrocytes, and no staining was found in hypertrophic chondrocytes. Osteoblasts along the trabeculae or periosteum displayed a strong staining intensity (Figure 1I–1K).

The 2 wk post-fracture time point corresponds to the hard callus stage when hypertrophic cartilage is being actively replaced by woven bone. Hypertrophic chondrocytes did not exhibit staining. Osteoblasts either lining the periosteum or along the islands of woven bone within the provisional callus also showed strong staining signal. However, the intensity of staining became fainter as osteoblasts matured to osteocytes (Figure 1L–1N).

The callus was composed primarily of bone 3 wk after the fracture, and cartilage was barely detected. *LacZ* staining was detected in osteoblasts but there was a very low level of staining in the fully differentiated osteocytes (Figure 1O–1Q). The 5 wk post-fracture time point corresponds to the bone remodeling phase, and the callus was composed of bone undergoing remodeling. At this time point, *LacZ* staining was mainly detected in osteoblasts residing in periosteum both adjacent to and farther from the fracture site (Figure 1R–1T).

In addition, since background *LacZ* staining can occur in wild-type animals, we also performed this staining on wild-type mice, and we failed to detect positive *LacZ* staining signals during fracture healing.

These observations suggest that β-catenin-mediated, TCF-dependent transcription is activated in both bone and cartilage formation during fracture healing. In particular, during endochondral ossification, β-catenin signaling is activated at the early stages of chondrogenesis, but not at later stages of chondrogenesis (i.e., hypertrophic chondrocytes). During osteogenesis, β-catenin is activated in cells with an osteoblast phenotype, but is down-regulated when osteoblasts undergo maturation into osteocytes.

### Precise Regulation of β-Catenin Signaling Is Required for Fracture Healing

**Figure 2:**
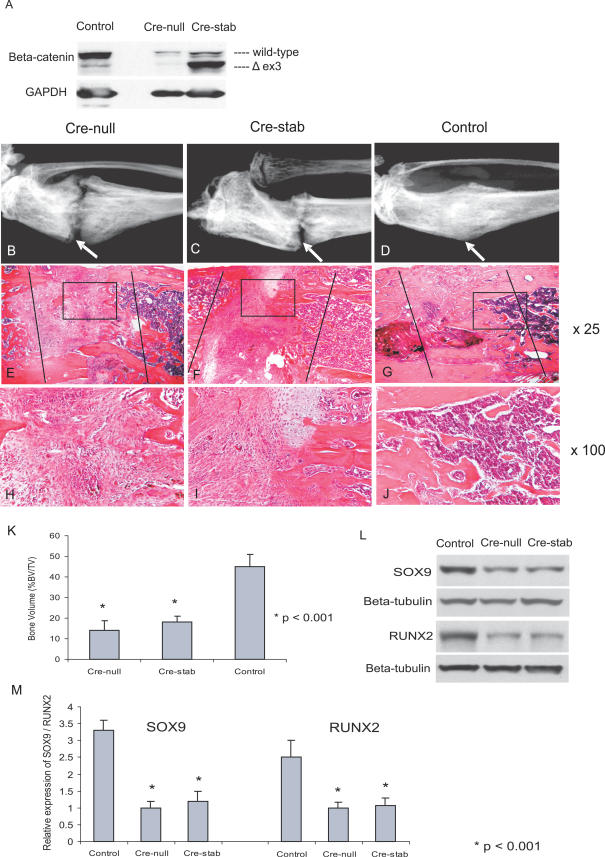
β-Catenin Loss- or Gain-of-Function during the Initial Repair Phase Represses Fracture Healing Ad-Cre and Ad-GFP (control) viruses were injected into fractures of *Catnb^lox(ex3)^* or *Catnb^tm2Kem^* (Cre-null and Cre-Stab, respectively) transgenic mice. (A) Regulation of β-catenin protein by Cre recombinase-mediated recombination of β-catenin alleles was confirmed by Western blot analysis of the fracture callus, which was harvested at 3 wk after the fracture. The stabilized mutant β-catenin protein is slightly smaller due to deletion of exon 3. (B–D) Radiographic analysis from Ad-Cre-treated *Catnb^tm2Kem^* mice (B), Ad-Cre-treated *Catnb^lox(ex3)^* mice (C), and Ad-GFP-treated control mice (D). At 3 wk after fracture, a lack of healing was seen in the mice expressing the null and stabilized alleles. Arrows indicate fracture site. (E–J) HE staining from Ad-Cre-treated *Catnb^tm2Kem^* mice (E and H); Ad-Cre-treated *Catnb^lox(ex3)^* mice (F and I), and Ad-GFP-treated control mice (G and J). The fracture sites consisted mainly of undifferentiated mesenchymal-like tissues in mice expressing either the null or stabilized β-catenin alleles, and there was still less remaining cartilage present in *Catnb^lox(ex3)^* mice at 3 wk after fracture. Histomorphometric analysis was conducted to analyze bone regeneration. Photomicrographs in the center row (E, F, and G) are magnified 25×; lines show the proximal and distal aspects of the healing fracture site. Images in the bottom row (H, I, and J) are 100× magnifications of areas shown in the boxes in the lower-magnification images. (K) Bone volume (BV) is expressed as a percentage of total callus tissue volume (TV). (L) Western blot of SOX9 at 1 wk and RUNX2 at 3 wk following fracture shows a decrease of both markers in the Cre-null and Cre-stab mice. (M) Relative expression of SOX9 and RUNX2 were quantified using densitometry;

To determine the role of β-catenin during fracture healing, we utilized a loss-of-function and gain-of-function approach. The repair process was observed in *Catnb^tm2Kem^* or *Catnb^lox(ex3)^* mice, which conditionally express null or stabilized β-catenin alleles, respectively, when subjected to Cre recombinase [25,26]. Ad-Cre was injected into the fracture site to promote the recombination of the conditional alleles. Ad-GFP was used as a control. Western blot analysis on protein from the fracture callus showed that treatment with Ad-Cre resulted in striking regulation of β-catenin expression (Figure 2A). To study the effect of the virus itself on fracture repair, we generated tibia fractures in wild-type mice and found no radiographic or histological difference in the healing phenotype after injection with either the Ad-Cre or the Ad-GFP control virus

At 3 wk following fracture, radiographic examination showed no evidence of bone bridging the fracture site in *Catnb^tm2Kem^* mice treated with Ad-Cre, and there was little new bone formation (Figure 2B). To our surprise, in the *Catnb^lox(ex3)^* mice, in which gene encoding β-catenin was conditionally stabilized after treatment with Cre recombinase, radiographic analysis also did not show evidence of bone bridging the fracture site (Figure 2C). Upon histological examination, fracture sites in *Catnb^tm2Kem^* mice mainly consisted of undifferentiated mesenchymal-like tissues, with no osteoblasts detected. *Catnb^lox(ex3)^* mice, however, showed a surprisingly similar histological appearance to that in *Catnb^tm2Kem^* mice, although less remaining cartilage was observed (Figure 2E–2J). Histomorphometric measurements further confirmed the inhibition of bone regeneration in these two transgenic mice (Figures 2K and S1).

We also observed a significant down-regulation of SOX9 and RUNX2 in both of these mice (Figure 2L and 2M). SOX9 is a transcription factor for chondrogenic differentiation, and is expressed in chondroprogenitors and chondrocytes [33,34]. RUNX2 is a transcription factor up-regulated during osteoblast differentiation, and is also expressed by some hypertrophic chondrocytes [35]. The down-regulation of these two transcription factors, in agreement with our histological findings, suggests a lack of both chondrogenic and osteogenic differentiation at the fracture sites in these two transgenic mice.

### β-Catenin Is Regulated by WNTs during Fracture Repair

Since β-catenin can be regulated by WNT ligands, we examined the expression of WNTs and their receptors in the fracture callus using RT-PCR. Many WNT ligands were expressed during fracture healing, including WNT4, 5b, 10b, 11 and 13. Furthermore, the receptors FZ1, 2, 4, and 5, and LRP6 were also up-regulated during fracture healing (Figure S2). Since WNT4 and WNT10b could mediate canonical WNT signaling (WNT/β-catenin pathway) [36,37], it is likely that these WNT ligands are responsible for activating β-catenin signaling during fracture repair. Furthermore, we also observed an up-regulation of WNT5a and protein kinase C alpha (PKCα) during fracture repair using Western blot analysis (Figure S2). Since WNT5a can mediate WNT/Ca^2+^ pathway signaling through its mediator PKCα [38], our results imply that the WNT signaling pathway is complex during fracture healing, likely involving activation of both canonical and noncanonical WNT pathways.

**Figure 3:**
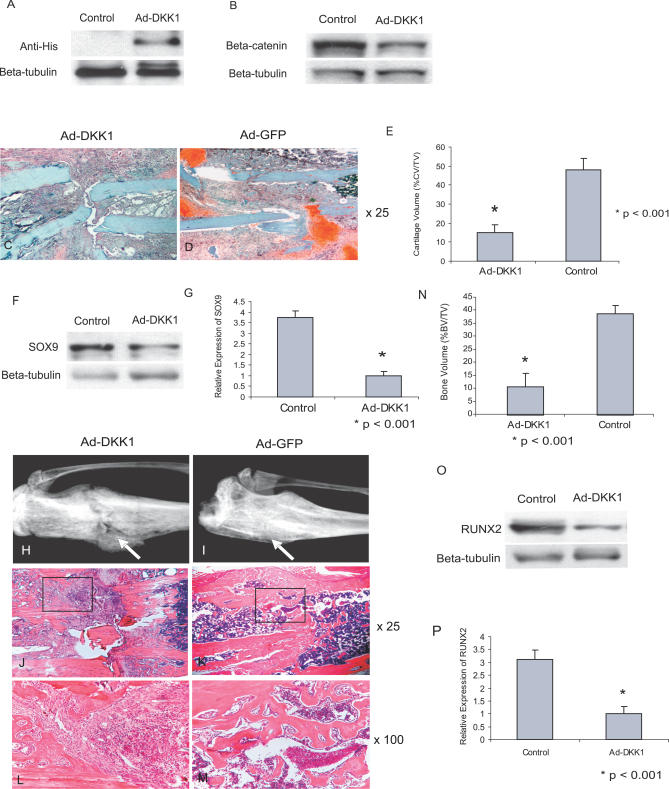
WNT Ligands Regulate β-Catenin during Fracture Healing (A) Western blot analysis shows expression of DKK1 as detected by an antibody to the His tag. (B) β-catenin was substantially down-regulated in fractures treated with DKK1. (C and D) SO staining of fracture samples from DKK1-treated mice (C) and control mice (D) at 1 wk after fracture show that DKK1 treatment down-regulates chondrogenic differentiation. Images are magnified 25×. (E) Histomorphometric analysis shows a down-regulation of cartilage volume (CV) at 1 wk after fracture, as a percentage of total callus tissue volume (TV), in DKK1-treated mice. (F and G) SOX9 was also down-regulated after treatment with Ad-DKK1 at 1 wk following fracture. The expression data are consistent with our histological findings. (H and I) Radiographic analysis from DKK1-treated mice (H) and from control mice (I), at 3 wk after fracture, shows a lack of healing in the DKK1-treated animals. Arrows indicate fracture site. (J–M) HE staining from DKK1-treated mice (J and L) and from control mice (K and M) undifferentiated mesenchymal-like cells at the fracture site in DKK1-treated animals at 3 wk after fracture. Images (J and K) are magnified 25×; (L and M) show 100× magnifications of the area shown in the box in the lower-magnification images. (N) Assay of bone volume (BV) as a percentage of total callus tissue volume (TV) shows a significant decrease in bone regeneration after DKK1 treatment. (O and P) RUNX2 was also significantly down-regulated in DKK1-treated mice, suggesting an inhibition of osteoblastic differentiation.

To investigate the contribution of WNT ligands to β-catenin regulation in fracture repair, we used an adenovirus expressing a His-tagged DKK1, an antagonist of canonical WNT/β-catenin signaling that inhibits WNT receptor activation [39,40]. Ad-GFP was also injected into the fracture site as a control. DKK1 expression was detected by Western blot analysis using anti-His antibody 1 wk following fracture (Figure 3A). Callus samples from animals treated with Ad-DKK1 exhibited a substantial reduction of β-catenin protein level, as compared to those injected with control virus (Figure 3B), confirming that WNT ligands regulate β-catenin during fracture repair.

At 1 wk after the fracture, SO staining and histomorphometric analysis showed that almost no cartilage islands were present in mice treated with DKK1, as compared to control animals (Figure 3C–3E). SOX9 was also down-regulated after treatment of Ad-DKK1, suggesting an inhibition of chondrogenic differentiation after blocking of WNT/β-catenin (Figure 3F and 3G). At 3 wk after the fracture, all of the control animals showed complete healing, while in DKK1-treated mice, fractures failed to heal (Figure 3H and 3I). There was a substantial reduction of both bone and cartilage volume in DKK1-treated mice, and the fracture site was mainly filled with large amount of undifferentiated mesenchymal-like tissues (Figure 3J–3M). Histomorphometric measurements confirmed this inhibition of bone formation (Figures 3N and S1). These phenotypes were nearly identical to the results in mice expressing Cre-mediated conditional β-catenin null alleles, suggesting a substantial inhibition of bone healing by inactivation of the WNT/β-catenin pathway. Furthermore, we examined the expression of RUNX2 in these mice, and observed that RUNX2 was expressed at substantially lower levels in mice expressing DKK1, as compared to control animals (Figure 3O and 3P).

### β-Catenin Plays a Disparate Role in Undifferentiated Mesenchymal Cells and Cells Committed to the Osteoblast Lineage

**Figure 4:**
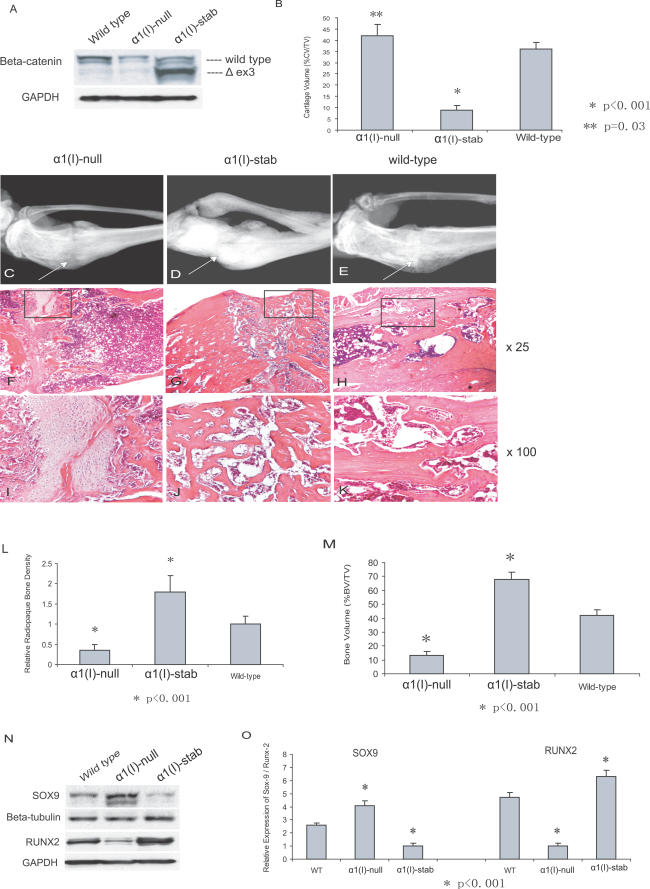
β-Catenin Acts to Regulate Bone Formation in Mice Expressing Osteoblast-Specific β-Catenin Null or Stabilized Alleles (A) Western blot analysis shows that β-catenin was regulated in the bone as expected in *α1(I)-Catnb^null^* and *α1(I)-Catnb^stab^* (*α1(I)-null* and *α1(I)-stab,* respectively) mice. (B) Histomorphometric analysis of calluses at 1 wk following fracture, as indicated by cartilage volume (CV) as a percentage of total callus tissue volume (TV). (C–E) Radiographic analysis from *α1(I)-Catnb^null^* mice (C), from *α1(I)-Catnb^stab^* mice (D), and from wild-type littermate mice (E) at 3 wk after fracture. Arrows indicate fracture site. (F–K) HE staining from *α1(I)-Catnb^null^* mice (F and I), from *α1(I)-Catnb^stab^* mice (G and J), and from wild-type mice (H and K) shows that bone healing was inhibited in *α1(I)-Catnb^null^* mice at 3 wk after fracture, with remaining cartilage at the fracture site. *α1(I)-Catnb^stab^* mice showed an accelerated fracture repair with abundant new bone tissue formed. Images (F, G, and H) are 25× magnifications; (I, J, and K) are 100× magnifications of the areas shown in the box in the lower-magnification images. (L) Relative radiopaque bone density at the fracture site shows decreased bone density in *α1(I)-Catnb^null^* mice and increased bone density in *α1(I)-Catnb^stab^* mice. (M) Assay of bone volume (BV) as a percentage of total callus tissue volume (TV) shows that *α1(I)-Catnb^null^* mice displayed inhibition of fracture healing, whereas osteoblast-specific β-catenin activation in *α1(I)-Catnb^stab^* mice enhanced bone healing. (N and O) Regulation of SOX9 at 1 wk and RUNX2 at 3 wk following fracture in *α1(I)-Catnb^null^, α1(I)-Catnb^stab^,* and wild-type mice.

To determine the function of β-catenin in osteoblasts, we observed fracture healing in mice in which β-catenin loss-of-function or gain-of-function was restricted to cells committed to the osteoblast lineage. Mice expressing conditional alleles were crossed with *α1(I)-Cre* mice (which restrict *Cre* expression to osteoblasts [27]), resulting in mice expressing the β-catenin null allele in osteoblasts [*α1(I)-Catnb^null^*] or β-catenin stabilized alleles in osteoblasts [*α1(I)-Catnb^stab^*]. A significant increase in bone mass was found in *α1(I)-Catnb^stab^* mutant mice, as previously reported [16]. Western blot analysis confirmed the expected changes in β-catenin protein level in the mice (Figure 4A)

At 1 wk following the fracture, *α1(I)-Catnb^null^* mice exhibited a fracture callus mainly composed of cartilage matrix, while chondrogenesis was not evident in *α1(I)-Catnb^stab^* mice (unpublished data). Histomorphometric assay confirmed these histology changes (Figure 4B). At 3 wk after the fracture, radiographic examination in *α1(I)-Catnb^null^* mice showed that, although there was some calcified callus present, the newly formed bone had not completely bridged the fracture gap, as compared to wild-type mice (Figure 4C and 4E). Radiographic quantification confirmed low bone density in *α1(I)-Catnb^null^* mice, as compared to wild-type mice (Figure 4L). Histological examination showed that the bone ends were not completely approximated in the *α1(I)-Catnb^null^* mice (Figure 4F and 4I). Histomorphometric analysis further showed a significant repression of bone regeneration in *α1(I)-Catnb^null^* mutant mice (Figures 4M and S1).

Surprisingly, radiographic examination in *α1(I)-Catnb^stab^* mice showed enhanced fracture healing, characterized by a calcified callus bridging the fracture gap as well as an increase in bone density (Figure 4D and 4E). Radiographic quantification, histological studies, and histomorphometric analysis further revealed that, compared to control animals, *α1(I)-Catnb^stab^* mutant mice showed a larger volume of regenerated bone tissues within the fracture site (Figures 4G, 4H, 4J, 4K–4M, and S1).

We also examined expression of SOX9 at 1 wk following fracture, and observed an up-regulation of this transcription factor in the *α1(I)-Catnb^null^* mutant mice. However, SOX9 was down-regulated in *α1(I)-Catnb^stab^* mutant animals. Expression of RUNX2 in these two mice, however, was regulated in the opposite manner (Figure 4N and 4O).

Since these conditional β-catenin alleles are regulated by a collagen type I promoter and thus are present in precursor cells committed to the osteoblast lineage but not in undifferentiated mesenchymal cells [27], our results support the concept that early osteoblast lineage cells lacking β-catenin are blocked in osteoblast differentiation and develop a chondrocyte phenotype instead. Expression of stabilized β-catenin in cells committed to the osteoblast lineage improves osteogenesis, thus leading to enhanced fracture healing.

### Lithium Treatment Enhances Healing if Started after the Fracture

**Figure 5:**
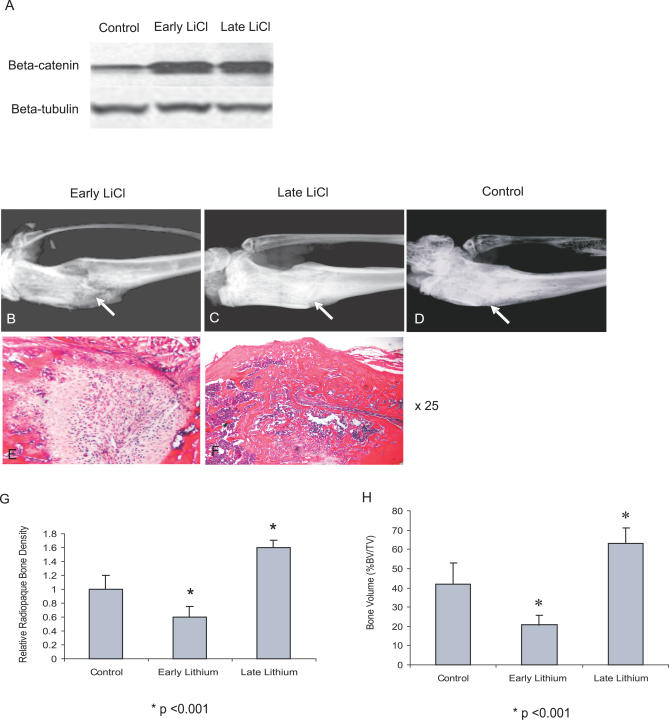
Lithium Treatment Regulates Bone Mass at the Fracture Site Mice were treated with lithium starting either 2 wk before the fracture (early treatment) or 4 d after the fracture (late treatment). (A) β-catenin increased 3 wk after fracture in mice receiving either early or late lithium treatment. (B–D) Radiographic analysis from mice receiving early lithium treatment (B), late lithium treatment (C), or NaCl as a control (D), show that lithium enhanced fracture healing only when given late. Arrows indicate the fracture site. (E and F) HE staining from mice receiving early or late lithium treatments, respectively (25×). There is immature mesenchymal tissue at the fracture site in (E), and large amounts of bone in (F). (G) Relative radiopaque bone density results show that early and late treatment have opposite effects on fracture healing. (H) Histomorphometric analysis on bone volume (BV) as a percentage of total callus tissue volume (TV), showing that pharmacologic activation of β-catenin after healing cells become committed to the osteoblast lineage could improve healing, while treatment at earlier time points inhibited fracture repair.

Lithium activates β-catenin signaling by inhibiting GSK3β [41–43]. Also, lithium enhances bone formation and improves bone mass in mice [31]. To asses the effect of lithium on fracture repair, treatment was started either 2 wk prior to initiating the fracture, or 4 d following the fracture in wild-type mice. Lithium treatment increased the level of β-catenin in the fracture tissue in both groups (Figure 5A). Mice in which the lithium treatment was started before the fracture had reduced bone in the fracture site, while mice in which the lithium treatment was started late displayed an enhanced fracture healing with relatively high radiopaque bone density (Figure 5B–5D). Histological examination of the fracture sites (Figure 5E and 5F) shows undifferentiated mesenchymal cells at the fracture site with early lithium treatment, while there is increased bone volume at the fracture site with late treatment. Radiographic and histomorphometric analysis further provided evidence that LiCl treatment enhances fracture healing, but only when utilized after the fracture (Figure 5G and 5H).

Lithium can have other cellular effects in addition to inhibiting GSK3β. To determine if lithium is indeed acting through β-catenin, we gave lithium treatment to *Catnb^tm2Kem^* mice in which the conditional null allele was activated by Ad-Cre. We found that mice expressing the null allele did not form as much bone at the fracture site as did wild-type mice or *Catnb^tm2Kem^* mice treated with lithium but not Ad-Cre (Figure S3), suggesting that lithium acts at least in part through β-catenin. Although data from the *α1(I)-Catnb^stab^* mice may be confounded by an increased bone density prior to the fracture, the results from lithium treatment shows that stabilization of β-catenin through a pharmacologic approach initiated after the fracture results in a similar enhancement in bone healing. This suggests that the enhanced bone repair in *α1(I)-Catnb^stab^* mice is due to β-catenin levels in the fracture healing cells committed to the osteoblast phenotype in the healing fracture, rather than to the underlying increased bone mass alone.

Taken together, our results confirm that lithium increases bone healing through its activation of β-catenin signaling pathway. These data, therefore, not only support our notion that β-catenin signaling functions differently at different stages of fracture repair, but more importantly, raises the possibility that lithium treatment can be used to enhance bone repair.

## Discussion

In this study, we demonstrated that β-catenin signaling plays a crucial role in fracture healing. We observed that precise regulation of β-catenin is important in the early phases of fracture healing to allow differentiation of mesenchymal cells into osteoblast and chondrocyte lineages. Our findings also indicate that β-catenin plays a disparate role in undifferentiated mesenchymal cells and in committed osteoblasts, and as such acts differently during different phases of fracture repair. These findings are important not only for understanding the role of β-catenin in different cell types, but also has a practical implication for therapy—pre-fracture lithium treatment inhibits the repair process, but post-fracture treatment enhanced bone healing. As such, lithium will enhance fracture healing, but only if started after cells have become committed to the osteoblast lineage.

During fracture repair, β-catenin-mediated TCF-dependent transcription is not activated in the earliest mesenchymal tissues present at the fracture site. Once cells that begin to show phenotypic features of either chondrocyte or osteoblast precursors, they exhibit β-catenin-mediated TCF-dependent transcription activity, suggesting a role in the initial phases of differentiation. In osteoblast lineage cells, β-catenin signaling activity decreased in later phases of differentiation, as osteoblasts matured into osteocytes. This finding is in agreement with a previous in vitro study, in which van der Horst and colleagues also observed a down-regulation of β-catenin signaling during the formation of a mineralized bone matrix in KS483 cells undergoing late phase osteoblast differentiation [44], suggesting a similar role for β-catenin in osteoblasts in fracture healing. During limb development, β-catenin is expressed in prechondrogenic mesenchymal cells but is significantly decreased in differentiated chondrocytes [45]. However, in our study, we found that β-catenin-mediated TCF-dependent transcription was also activated in chondrocytes and, to a lesser extent, in prehypertrophic chondrocytes. Thus, the range of activity for β-catenin mediated TCF-dependent transcription in chondrocytes is greater in fracture healing than in development, suggesting that β-catenin plays a role in chondrocytes over a broader range of differentiation in repair than in development.

Our results showed that many WNT ligands and receptors are selectively up-regulated during bone healing, several of which (e.g., WNT4, WNT10b, and LRP6) are known to activate the canonical WNT pathway [36,37]. Moreover, our data showing that DKK1 treatment inhibits β-catenin signaling and suppresses fracture repair suggest that the canonical WNT pathway is the dominant mechanism regulating β-catenin during bone healing. Since multiple WNT ligands that activate β-catenin signaling are expressed during the repair process, there is likely functional redundancy. However, our results do not exclude the possibility that noncanonical WNT pathways are also likely activated during fracture repair, as WNT5a and WNT11 can signal through the WNT/Ca^2+^ pathway, which regulates Ca^2+^ flux and Ca^2+^-sensitive protein kinases and transcription factors, such as PKCα and calcium/calmodulin-dependent protein kinase II [38,46]. WNT11 can also activate the c-Jun N-terminal kinase pathway [47,48]. Indeed, in our study, we not only noticed that WNT11 was expressed during fracture healing, but also observed an activation of both WNT5a and its mediator PKCα. These results suggest that the role for WNT ligands in fracture repair is complex, potentially involving activation of multiple pathways (i.e., canonical and noncanonical WNT pathways).

We observed that β-catenin-mediated TCF-dependent transcription is activated during both chondrogenesis and osteogenesis in fracture repair. Mice expressing conditional β-catenin null alleles after treatment of Cre recombinase failed to heal, and there was a lack of bone and cartilage at the fracture site. The histology studies showed immature mesenchymal cells at the fracture sites. This finding is not unexpected given the role of β-catenin during fetal bone development, in which conditional deficiency in early osteoblast progenitors results in a blockade of osteoblast differentiation, with immature mesenchymal cells persisting [49,50]. However, fractures also failed to heal in *Catnb^lox(ex3)^* mice whose β-catenin-encoding gene had been stabilized by treatment with Cre recombinase. The fracture site in this case was also composed of immature mesenchymal cells, suggesting a block to differentiation. This observation was in stark contrast to the results from *α1(I)-Catnb^stab^* mice, which expressed osteoblast-specific stabilized β-catenin alleles; these mice exhibited significantly enhanced bone healing. Thus, during fracture healing, Cre-mediated conditional β-catenin stabilization represses differentiation of these mesenchymal cells, perhaps maintaining these cells in an undifferentiated proliferative state. Previous in vitro studies and studies of bone development provide evidence to support this notion, as WNT signaling activation maintains mesenchymal stem cells in a less differentiated state during osteogenic differentiation, and stabilization of β-catenin can interfere with the differentiation of precursors into chondrocytes as well as osteoblasts during development [49–51]. In cells that have already adopted an osteoblast phenotype, β-catenin signaling activation works differently; that is, it promotes osteoblastic differentiation and enhances osteogenesis. Therefore, the level of β-catenin needs to be precisely regulated at the earliest phase of fracture healing to allow for proper differentiation.

Interestingly, fractures in *α1(I)-Catnb^null^* mice healed with abundant cartilage matrix at the fracture site, while *Catnb^tm2Kem^* mice in which conditional β-catenin null alleles were regulated by Ad-Cre mainly exhibited undifferentiated mesenchymal-like cells at the fracture site. These data imply that mesenchymal precursors that have already become committed to an osteoblast phenotype will adopt a chondroblastic phenotype instead if β-catenin is absent. This result agrees with data from bone development, which show that osteoblast precursors lacking β-catenin are blocked in differentiation and develop into chondrocytes instead [49,50].

Our findings raise the possibility that therapy to activate β-catenin could be used to enhance fracture repair. However, since activation of β-catenin signaling inhibits differentiation of mesenchymal cells not yet committed to the osteoblastic phenotype, such treatment should be utilized only after cells are committed to the osteoblast lineage. One potential therapeutic agent is lithium, which activates the canonical WNT signaling through inhibition of GSK3β [41–43], and has demonstrated its capacity to increase bone formation and improve bone mass in mice [31]. We found that lithium treatment greatly up-regulates β-catenin, and indeed has the potential to enhance fracture healing, but only if started several days after the fracture has occurred. Since lithium is a pharmacologic agent, and oral lithium has been used to treat humans with bipolar disease for over a half-century with substantial benefit [52], its use to improve bone repair could readily be tested in the clinical setting.

## Supporting Information

Alternative Language Abstract S1 Translation of the Abstract into Mandarin by Yan Chen (144 KB PDF)

Figure S1 Detailed Histomorphometric Data For histomorphometric analysis, callus tissues at 3 wk after fracture were fixed in 4% paraformaldehyde, decalcified in 20% EDTA (pH 7.4), and embedded in paraffin. 10 μm sections were prepared and stained with HE. For each callus, an average of ten tissue sections was used to determine callus parameters, including trabecular thickness (μm), trabecular number (per mm), and trabecular separation (μm). Four animals were analyzed for each group. Data were expressed as mean ± standard deviation. Statistical differences were calculated by using Student *t-*test. *p* < 0.001 was considered statistically significant (*p < 0.001).
(A) Trabecular number assay from Ad-Cre-treated *Catnb^tm2Kem^, Catnb^lox(ex3)^,* and Ad-GFP-treated control mice.
(B) Trabecular thickness assay from Ad-Cre-treated *Catnb^tm2Kem^, Catnb^lox(ex3)^,* and Ad-GFP-treated control mice.
(C) Trabecular separation assay from Ad-Cre-treated *Catnb^tm2Kem^, Catnb^lox(ex3)^,* and Ad-GFP-treated control mice.
(D) Trabecular number assay from Ad-DKK1- and Ad-GFP-treated wild-type mice.
(E) Trabecular thickness assay from Ad-DKK1- and Ad-GFP-treated wild-type mice.
(F) Trabecular separation assay from Ad-DKK1- and Ad-GFP-treated wild-type mice.
(G) Trabecular number assay from *α1(I)-Catnb^null^, α1(I)-Catnb^stab^,* and wild-type mice.
(H) Trabecular thickness assay from *α1(I)-Catnb^null^, α1(I)-Catnb^stab^,* and wild-type mice.
(I) Trabecular separation assay from *α1(I)-Catnb^null^, α1(I)-Catnb^stab^,* and wild-type mice.
(781 KB PDF)

Figure S2 WNT-Related Gene Expression At different time points after fracture, calluses were harvested and total RNA was isolated. Expression of mRNA was compared to that of the housekeeping control β-2 macroglobin. All experiments were performed in triplicate. Protein extracts were also isolated, and Western blot analysis was performed to determine protein expression. Protein expression was also normalized to GAPDH as a loading control.
(A) Several WNT ligands (WNT4, 5b, 10b, 11, and 13) and receptors (FZ1, 2, 4, and 5, and LRP6) were activated at mRNA level during fracture healing.
(B) Both WNT5a and its signaling mediator PKCα were up-regulated during fracture repair.
(737 KB PDF)

Figure S3 The Effect of Lithium Treatment on Bone Density is Partially Mediated by β-Catenin Bone healing was observed 3 wk following generation of a tibia fracture in mice treated with lithium or control started after creation of the fracture. There were five mice in each group. In mice expressing β-catenin null alleles activated by treatment with Ad-Cre there was reduced bone mass compared to mice in which Ad-Cre was not administered or mice in which neither Ad-Cre nor lithium was administered. An asterisk above the mean indicates a statistically significant difference between β-catenin null and wild-type mice treated with lithium. Control mice (not treated with lithium) were assigned an average relative bone density of 1. These data show that the effect of lithium is at least partially mediated by β-catenin.
(2.6 MB PDF)

Table S1 PCR Primer Sequences (781 KB PDF)

Table S2 Antibodies Used in This Study (781 KB PDF)

## Abbreviations

DKK: dickkopf
DVL: dishevelled
FZ: frizzled
GAPDH: glyceraldehyde 3-phosphate dehydrogenase
GSK3β: glycogen synthase kinase 3 beta
HE: hematoxylin–eosin
LEF: lymphoid enhancer factor
LRP: low-density lipoprotein jureceptor-related protein
PKCα: protein kinase C alpha
siRNA: small interfering RNA
SO: safranin O
TCF: T cell factor
WISP1: WNT1-induced secreted protein 1
