# Supplementary material for: Beta-Catenin Signaling Plays a Disparate Role in Different Phases of Fracture Repair: Implications for Therapy to Improve Bone Healing
Source: PLoS Med. 2007 Jul 31;4(7):e249. doi: 10.1371/journal.pmed.0040249 (PMC1950214; doi:10.1371/journal.pmed.0040249)
Supplement: Alternative Language Abstract S1 — (144 KB PDF) [file pmed.0040249.sd001.pdf]

Beta-catenin 信号传导通路在骨折愈合中的作用及其治疗意义

研究背景

骨折延迟愈合是临床中的难题，如果能利用药物来促进愈合则会改善骨折病人的预后。信号传导通路在骨折愈合中的作用已经得到日益广泛的重视。通过对这些不同的信号通路的研究可以有助于了解骨折愈合的分子机理，并为未来的药物治疗创造基础。β-catenin 介导的 Tcf (T cell factor, T 细胞因子) 依赖性信号传导已经被证实能调节成骨细胞的增值分化，并在胚胎骨骼发育中发挥关键的调控作用。然而，β-catenin 信号传导在骨再生过程中的作用尚未见报道。

实验方法与结果

我们在小鼠胫骨建立骨折模型，以此观察骨折愈合效果。应用 Western blot 分析，发现 β-catenin 蛋白表达水平在骨折愈合中显著提高；利用 β-gal 染色发现在 Tcf 转基因小鼠骨折愈合过程中，β-catenin 介导的 Tcf 依赖性信号传导通路被显著激活；逆转录 PCR 分析结果表明多个 Wnt 配体分子在愈合过程中高表达，提示 β-catenin 信号通路很可能被 Wnt 配体所激活。对小鼠骨折部位注射表达 Dkk-1 (Wnt/β-catenin 信号通路拮抗剂) 的腺病毒载体后发现骨折愈合被明显延迟，这进一步说明骨折愈合过程中 β-catenin 信号通路的激活直接受来自 Wnt 配体分子的调控。另外，我们在两种不同的含有 Cre/loxp 系统的 β-catenin 转基因小鼠 (*Knock-out* 及 *Knock-in*) 骨折部位注射 Cre 重组酶腺病毒载体后，发现骨折愈合在这两种小鼠体内均被抑制。进一步研究发现，骨折愈合在表达成骨细胞特异性的 β-catenin 基因 *Knock-out* 小鼠体内也被抑制。与此形成鲜明对照的是，在表达成骨细胞特异性的 β-catenin 基因 *Knock-in* 转基因小鼠体内，骨折愈合得以显著增强。这些结果表明成骨细胞内激活 β-catenin 信号通路可以促进骨折愈合。以此为理论依据，我们进一步发现，应用 β-catenin 信号激活剂 LiCl 来调节骨折愈合会产生不同的效果：如果术前两周开始给予口服 LiCl 治疗，骨折愈合被抑制；但如果术后四天开始给予 LiCl 治疗，则可以显著促进骨折愈合。

结论

研究结果表明，β-catenin 信号通路在骨折不同愈合阶段发挥不同的作用。在愈合起始阶段，β-catenin 信号通路受到严密调控以使得具有多分化潜能的间充质细胞分化为成骨细胞或软骨细胞。一旦这些未分化的间充质细胞具备了早期成骨细胞表型，β-catenin 信号传导的激活则能发挥对成骨细胞的正向调节作用，并增进骨折愈合。β-catenin 这一作用与其在胚胎骨骼发育中的作用是有区别的。应用 LiCl 激活 β-catenin 信号通路可以有效增强骨折愈合，但只有在未分化间充质细胞分化为成骨细胞表型后才能发挥这种促进作用。

Translation of the abstract into language MANDARIN by author YAN CHEN
