# Supplementary figures and images for: Beta-Catenin Signaling Plays a Disparate Role in Different Phases of Fracture Repair: Implications for Therapy to Improve Bone Healing
Source: PLoS Med. 2007 Jul 31;4(7):e249. doi: 10.1371/journal.pmed.0040249 (PMC1950214; doi:10.1371/journal.pmed.0040249)

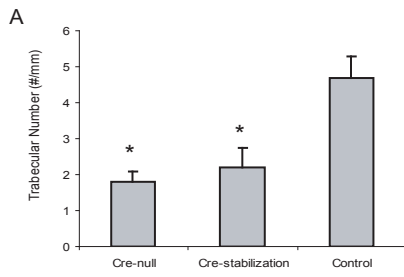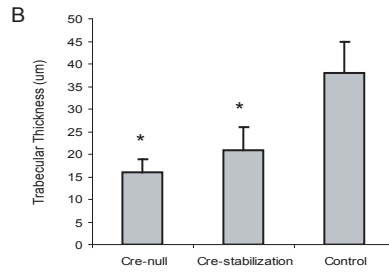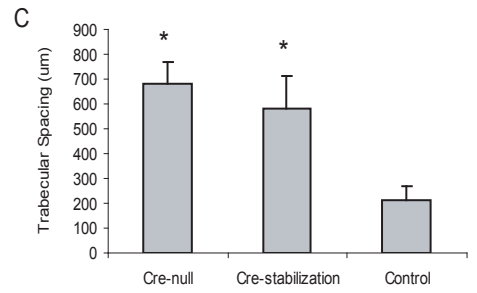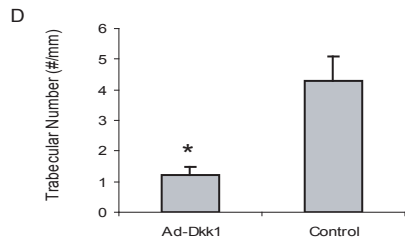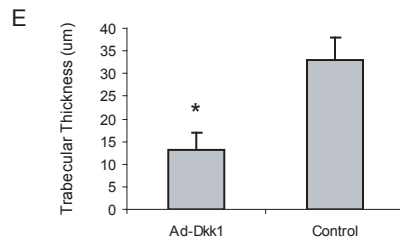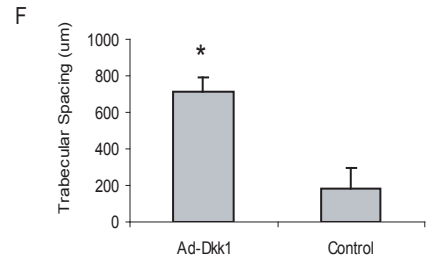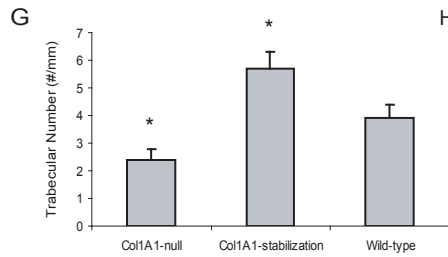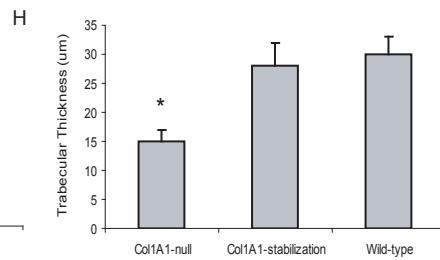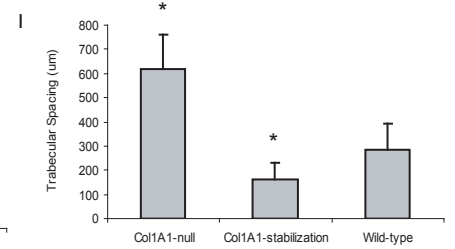

Supplement: Figure S1 — For histomorphometric analysis, callus tissues at 3 wk after fracture were fixed in 4% paraformaldehyde, decalcified in 20% EDTA (pH 7.4), and embedded in paraffin. 10 μm sections were prepared and stained with HE. For each callus, an average of ten tissue sections was used to determine callus parameters, including trabecular thickness (μm), trabecular number (per mm), and trabecular separation (μm). Four animals were analyzed for each group. Data were expressed as mean ± standard deviation. Statistical differences were calculated by using Student t-test. p < 0.001 was considered statistically significant (*p < 0.001). (A) Trabecular number assay from Ad-Cre-treated Catnbtm2Kem, Catnblox(ex3), and Ad-GFP-treated control mice. (B) Trabecular thickness assay from Ad-Cre-treated Catnbtm2Kem, Catnblox(ex3), and Ad-GFP-treated control mice. (C) Trabecular separation assay from Ad-Cre-treated Catnbtm2Kem, Catnblox(ex3), and Ad-GFP-treated control mice. (D) Trabecular number assay from Ad-DKK1- and Ad-GFP-treated wild-type mice. (E) Trabecular thickness assay from Ad-DKK1- and Ad-GFP-treated wild-type mice. (F) Trabecular separation assay from Ad-DKK1- and Ad-GFP-treated wild-type mice. (G) Trabecular number assay from α1(I)-Catnbnull, α1(I)-Catnbstab, and wild-type mice. (H) Trabecular thickness assay from α1(I)-Catnbnull, α1(I)-Catnbstab, and wild-type mice. (I) Trabecular separation assay from α1(I)-Catnbnull, α1(I)-Catnbstab, and wild-type mice. (781 KB PDF) [file pmed.0040249.sg001.pdf]

A

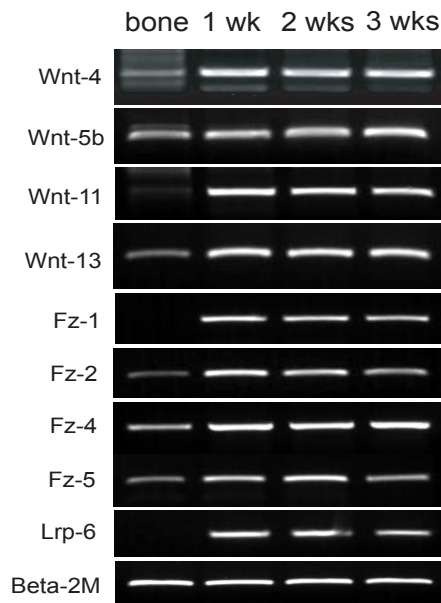

B

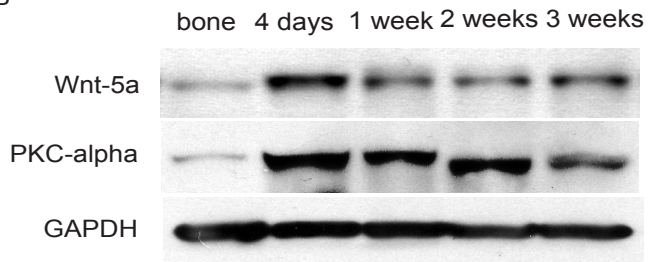

Supplement: Figure S2 — At different time points after fracture, calluses were harvested and total RNA was isolated. Expression of mRNA was compared to that of the housekeeping control β-2 macroglobin. All experiments were performed in triplicate. Protein extracts were also isolated, and Western blot analysis was performed to determine protein expression. Protein expression was also normalized to GAPDH as a loading control. (A) Several WNT ligands (WNT4, 5b, 10b, 11, and 13) and receptors (FZ1, 2, 4, and 5, and LRP6) were activated at mRNA level during fracture healing. (B) Both WNT5a and its signaling mediator PKCα were up-regulated during fracture repair. (737 KB PDF) [file pmed.0040249.sg002.pdf]

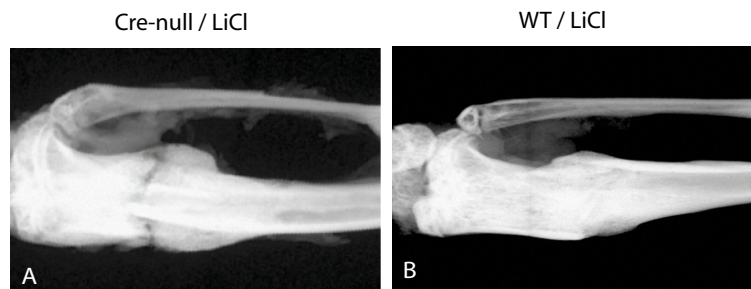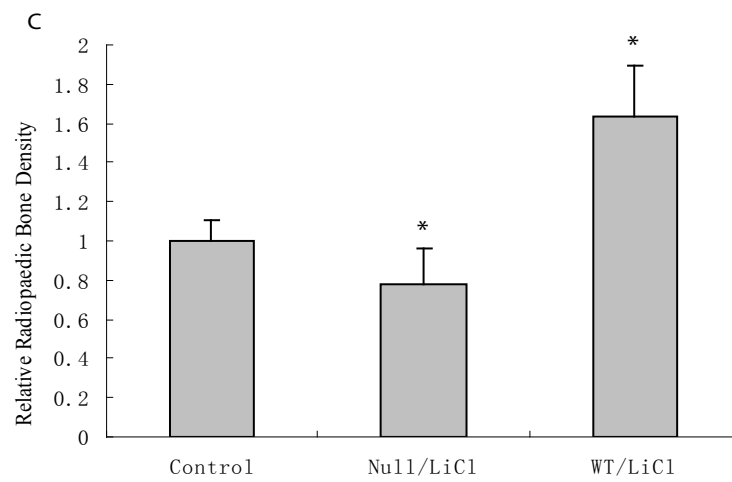

\*  $P < 0.05$

Supplement: Figure S3 — Bone healing was observed 3 wk following generation of a tibia fracture in mice treated with lithium or control started after creation of the fracture. There were five mice in each group. In mice expressing β-catenin null alleles activated by treatment with Ad-Cre there was reduced bone mass compared to mice in which Ad-Cre was not administered or mice in which neither Ad-Cre nor lithium was administered. An asterisk above the mean indicates a statistically significant difference between β-catenin null and wild-type mice treated with lithium. Control mice (not treated with lithium) were assigned an average relative bone density of 1. These data show that the effect of lithium is at least partially mediated by β-catenin. (2.6 MB PDF) [file pmed.0040249.sg003.pdf]
