# Supplementary material for: Beta-Catenin Signaling Plays a Disparate Role in Different Phases of Fracture Repair: Implications for Therapy to Improve Bone Healing
Source: PLoS Med. 2007 Jul 31;4(7):e249. doi: 10.1371/journal.pmed.0040249 (PMC1950214; doi:10.1371/journal.pmed.0040249)
Supplement: Table S1 — (781 KB PDF) [file pmed.0040249.st001.doc]

**Table S1** **PCR primers used**

| **Oligo Name** | | **Forward Sequence (5'-3')** | | |  | **Reverse Sequence (5'-3')** | |
| --- | --- | --- | --- | --- | --- | --- | --- |
|  |  |  |  |  |  |  |  |
| Wnt-4 |  | CTCAAAGGCCTGATCCAGAG | | | | TCACAGCCACACTTCTCCAG | |
| Wnt-5a |  | GTCTACCTGTGGCTGCAGC | | |  | GCACACAGTAGTCCGGACTG | |
| Wnt-5b |  | AGTGCAGAGACCGGAGATGT | | | | GACAGATGTGTTGTCCACGG | |
| Wnt-10b |  | GATACCCACAACCGCAACTC | | | | GGCTCACCTTCATTTACACACA | |
| Wnt-11 |  | GCTCCATCCGCACCTGTT | | |  | CGCTCCACCACTCTGTCC | |
| Wnt-13 |  | CACCCGGACTGATCTTGTCT | | |  | TGTTTCTGCACTCCTTGCAC | |
| Fz-1 |  | TGCCAGCCATCAAAACTATAAC | | | | AGAGGACACTGAAGACTCCG | |
| Fz-2 |  | CGGCTCTATGTTCTTCTCGC | | |  | AGCCGGACAGAAAGATGATG | |
| Fz-4 |  | GCTTCATCTCCACCACCTTC | | |  | TCAGTTCATCGGCATCCAC | |
| Fz-5 |  | ACCTGTGTGTGTCACTGGGA | | |  | ACTTGACACTGGGGATGAGC | |
| Lrp-6 |  | GGTGTCAAAGAAGCCTCTGC | | | | GCTCGAGGACTGTCAAGGTC | |
| β-catenin |  | GCGTGGACAATGGCTACTCAAG | | | | TATTAACTACCACCTGGTCCTC | |
| β-2M |  | AAATGCTGAAGAACGGGAAA | | | | GATGCTTGATCACATGTCTCG | |
