# Supplementary material for: Beta-Catenin Signaling Plays a Disparate Role in Different Phases of Fracture Repair: Implications for Therapy to Improve Bone Healing
Source: PLoS Med. 2007 Jul 31;4(7):e249. doi: 10.1371/journal.pmed.0040249 (PMC1950214; doi:10.1371/journal.pmed.0040249)
Supplement: Table S2 — (781 KB PDF) [file pmed.0040249.st002.doc]

**Table S2** **Antibodies used**

| **Antibody** |  |  |  | **Source** | **Manufacture** | | **Cat. No.** | |
| --- | --- | --- | --- | --- | --- | --- | --- | --- |
|  |  |  |  |  |  |  | |  |
| anti-β-catenin | |  |  | rabbit | Upstate |  | | 06-734 |
| anti-phospho-GSK-3β (Ser-9) | | | | rabbit | Cell Signaling | | 9336 | |
| anti-GSK-3β | |  |  | rabbit | Cell Signaling | | 9332 | |
| anti-β-tubulin | |  |  | mouse | Sigma |  | | T0198 |
| anti-Wnt-5a | |  |  | goat | R&D |  | | AF645 |
| anti-Runx-2 | |  |  | mouse | MBL |  | | D130-3 |
| anti-Sox-9 | |  |  | rabbit | abcam |  | | ab3697 |
| anti-GAPDH | |  |  | mouse | abcam |  | | ab9485 |
| anti-PKC-α | |  |  | mouse | BD Pharmingen | | 554207 | |
| HRP-conjugated anti-rabbit IgG | | | | goat | BD Pharmingen | | 554021 | |
| HRP-conjugated anti-mouse IgG | | | | goat | BD Pharmingen | | 554002 | |
